# Cytogenetic variation of repetitive DNA elements in *Hoplias malabaricus* (Characiformes - Erythrinidae) from white, black and clear water rivers of the Amazon basin

**DOI:** 10.1590/1678-4685-GMB-2015-0099

**Published:** 2016

**Authors:** Fabíola Araújo dos Santos, Diego Ferreira Marques, Maria Leandra Terencio, Eliana Feldberg, Luís Reginaldo R. Rodrigues

**Affiliations:** 1 Laboratório de Genética & Biodiversidade, Instituto de Ciências da Educação, Universidade Federal do Oeste do Pará, Santarém, PA, Brazil; 2 PPG Recursos Naturais da Amazônia, Universidade Federal do Oeste do Pará, Santarém, PA, Brazil; 3 Laboratório de Citogenética Animal, Instituto de Pesquisas da Amazônia, Manaus, AM, Brazil

**Keywords:** cytogenomics, FISH, rDNA, REX 3, Tapajós river

## Abstract

*Hoplias malabaricus* is a common fish species occurring in white, black and clear water rivers of the Amazon basin. Its large distribution across distinct aquatic environments can pose stressful conditions for dispersal and creates possibilities for the emergence of local adaptive profiles. We investigated the chromosomal localization of repetitive DNA markers (constitutive heterochromatin, rDNA and the transposable element REX-3) in populations from the Amazonas river (white water), the Negro river (black water) and the Tapajós river (clear water), in order to address the variation/association of cytogenomic features and environmental conditions. We found a conserved karyotypic macrostructure with a diploid number of 40 chromosomes (20 metacentrics + 20 submetacentrics) in all the samples. Heteromorphism in pair 14 was detected as evidence for the initial differentiation of an XX/XY system. Minor differences detected in the amount of repetitive DNA markers are interpreted as possible signatures of local adaptations to distinct aquatic environments.

## Introduction

The rivers in the Amazon basin, South America, are usually categorized based on the appearance of their water color type. Traditionally, these rivers are recognized as white, clear and black water types (Goulding, 1997). This classification system has been formally considered since the 1950s and is supported based on differences in water chemical properties, with white water rivers (*e.g.* Amazonas) having a neutral pH and turbid water, clear water rivers (*e.g.* Tapajós) a pH variable between 5 to 8 and a greenish water color, and black water rivers (*e.g.* Negro) having an acidic pH of < 5 and a brownish water color (Sioli, 1984).

An enormous diversity of fishes and other aquatic organisms inhabits the Amazon basin, where they interact with equally diverse, heterogeneous and dynamic habitats (Val and Almeida-Val, 1995). Genetic divergence is also expected from populations occurring in such distinct environment conditions. Farias and Hrbek (2008), observed restriction of gene flow between 'brown' discus and 'Heckel' discus (*Symphysodon*) populations from the Amazonas and Negro rivers, which was interpreted as result of local adaptation to distinct environments.

The understanding of how fish genomes interact to provide local adaptations to specific environmental conditions and their fluctuation is a frontier of scientific knowledge. Repetitive DNA represents a large portion of fish genomes and may play a role in genome structure maintenance, chromosomal physiology and possibly local adaptations processes (Charlesworth *et al.*, 1994; Burt and Trivers, 2006). Such genomic elements are observed as tandem arrays (minisatellite and microsatellite sequences) or dispersed repeats (transposons and retrotransposons) (Martins, 2007).

It is noteworthy that transposable elements mobility can be induced by environmental factors that contribute as source of new genetic variability that can be useful in the face of stressful conditions (Capy *et al.*, 2000). Gross *et al.* (2009), have detected variation in the genomic organization of retroelement *Rex 3* in *Symphysodon spp.* from distinct Amazon rivers. Such variation has been tentatively explained as local adaptations to water chemistry conditions. Dramatic transcriptomic response and genome sequence variation have been demonstrated in the Killifish (*Fundulus heteroclitus*) subjected to acclimation to extreme osmotic shock and pollution tolerance (Whitehead *et al.*, 2011).

Fish genomes are rich in all kinds of transposable elements (TEs) (Volff *et al.*, 2000). Physical mapping of TEs in different orders of fishes revealed a clustered pattern in Tetraodontiformes, but was variable in Perciformes, with several species exhibiting a dispersed pattern mode (Martins, 2007). The non LTR-retrotransposons *Rex 1* and *Rex 3* are abundant in teleost genomes, and have a predisposition to play a role in genome evolution and karyotype reorganization (Volff*et al.*, 1999; 2000; Ozouf-Costaz *et al.*, 2004; Gross *et al.*, 2009). A clustered distribution of*Rex3* has been found in *Astronotus ocellatus*(Mazzuchelli and Martins, 2009) and*Symphysodon spp*. (Gross*et al.*, 2009), both groups being native from the Amazon basin (Nelson, 2006). Interestingly, in the former, *Rex3* is co-localized with centromeric heterochromatin of all the chromosomes, while in the latter the accumulation of*Rex3* is preferentially in a few pairs. If the variation in the amount and/or distribution of TE in the genome of Amazonian fishes has a correlation with local adaptation is not clear.

In order to contribute to the knowledge of genome structure of Amazonian fishes we mapped the chromosomal localization of repetitive DNA elements in the karyotype of*Hoplias malabaricus* (Characiformes – Erythrinidae). This species is widespread throughout the Amazonian basin, occurring naturally in white, black and clear water environments. *H. malabaricus* is believed to harbor a species complex with seven distinct cytotypes, designated by letters A-G, and distinguished by clear chromosomal features, such as diploid number, presence/absence of sexual chromosome system and chromosome morphology (Bertollo *et al.*, 2000).

## Materials and Methods

### Sampling and chromosome preparation

We collected 60 specimens of *Hoplias malabaricus* from the Amazonas river (white water environment; n = 20), Tapajós river (clear water environment; n = 20) and Negro river (black water environment; n = 20) (for details see Table 1). Taxonomic identification was provided by examination of external characters followingOyakawa and Mattox (2009). Vouchers were fixed in formalin 10%, preserved in ethanol 70% and stored in the Fish Collections of Instituto Nacional de Pesquisas da Amazônia (INPA) and Universidade Federal do Oeste do Pará (UFOPA).

**Table 1:** Sampling localities of *Hoplias malabaricus* from Amazon basin.

River	Aquatic Environment	Exact sites	GPS	Field number of vouchers*
Amazonas	White water	Comunidade Camaleão, Almeirim-PA; Rio Amazonas, Santarém-PA	S 1° 31' 13.2″/ W 52° 33' 34.1″ S 2° 24' 8.8″/ W 54° 42' 51.2″	AMA-1(m), AMA-2(m), AMA-4(f), AMA-5(m), AMA −6(f), AMA-7(f), AMA-13(f), AMA-14(m), AMA-15(m), AMA-16(m), AMA-17(f), AMA-18(m), ALM-2(m), A LM-5(f), ALM-6(m), ALM-8(m), ALM-9(f), ALM-10(f), ALM-11(f), ALM-12(f)
Tapajós	Clear water	Lago Bem bom, Miritituba-PA; Lago Juá, Santarém-PA	S 4° 18' 28.8″/W 55° 59' 0.9″ S 2° 26' 40.0″/W 54° 47' 21.1″	JUA-9(m), JUA-13(f), JUA-14(m), JUA-15(m), ITA-47(m), ITA-48(f), ITA-66(f), ITA-70(m), ITA-71(m), IT A-102(m), ITA-104(f), ITA-105(m), ITA-107(m), ITA-1 09(m), ITA-110(m), ITA-112(f), ITA-113(m), ITA-114(m), ITA-116(m), ITA-117(m)
Negro	Black water	Lago Catalão, Manaus-AM	S 3° 09' 47″ / W 59° 54' 29″	10195(?), 10224(?), 10244(?), 10409(m), 10411(m), 11121(m), 11122(m), 11123(f), 11124(m), 11125(m), 11 126(m), 11127(m), 11128(f), 11129(m), 11130(m), 1113 1(m), 11132(f), 11133(m), 11134(m), 11135(m)

* In parentheses, sex of each individual, (m) for male and (f) for female (?) not identified.

Metaphasic chromosomes were obtained from kidney cells extracted in the field after treatment with colchicine 0.0025% in a dosage of 1 mL per 100 g of body mass (Bertollo *et al.*, 1978). The fishes were anesthetized with crave oil mixed with aquarium water before the tissue sampling.

### Karyotype and banding procedures

To determine diploid number we counted at least 30 metaphases that were conventionally stained with Giemsa diluted in phosphate buffer at 2%. At least five spreads were digitally photographed and the karyotypes organized using Adobe Photoshop version7. For chromosome nomenclature we followed Levan *et al.* (1964). Heterochromatic regions were detected with C-banding (Sumner, 1972). The Nucleolar Organizing Regions (NORs) were revealed by impregnation with silver nitrate following a standard protocol provided in Howell and Black (1980). Metaphases were stained with chromomycin A3 (CMA3) in order to detect GC rich bands, following the procedure of Schweizer (1980).

### DNA extraction, PCR and fluorescent in situ hybridization (FISH)

Genomic DNA was extracted from heart muscle tissue of *Hoplias malabaricus* following a standard phenol-chloroform protocol (Sambrook and Russell 2001).

Repetitive DNA probes were prepared by PCR using the primers cited in literature as follows: ***rDNA5S***- 5Sa (5-TAC GCC CGA TCT CGT CCG ATC) and 5Sb (5-CAG GCT GGT ATG GCC GTA AGC-3) Komiya and Takemura (1979); ***Rex3***- RTX3-F3 (5-CGG TGA YAA AGG GCA GCC CTG-3) and RTX3-R3 (5-TGG CAG ACN GGG GTG GTG GT-3), Volff *et al.*(1999; 2001).

The reactions were carried out in a final volume of 15 μl consisting of: 1 μl of genomic DNA of *Hoplias malabaricus* (200ng/μl); 1.5 μl buffer 10X; 2.4 μl of dNTP mix (1.25 mM); 0.6 μl MgCl2 (50 mM); 0.6 μl of each primer (5 mM); 0.12 μl *Taq* DNA polymerase (5 U/μl) and 8.18 μl of ultrapure water. The PCR profiles for **rDNA5S** was: 94 °C for 1 min, followed by 30 cycles of 94 °C for 1 min, 57 °C for 1 min, 72 °C for 90 s, an a final extension at 72 °C for 5 min. For ***Rex3*** it was: 95 °C for 2 min, followed by 35 cycles of 95 °C for 1 min, 55 °C for 40 s, 72 °C for 2 min, and a final extension at 72 °C for 5 min.

Positive reactions were visualized on 1% agarose gels stained with GelRed (Biotium-Uniscience). PCR products were used in a second reaction for probe labeling with bio-tin-14-dATP by nick translation with the BioNick Labeling System kit (Invitrogen) following the manufacturer's instructions. Hybridization reactions were carried out following the protocol described by Pinkel *et al.* (1986) with minor modifications. The slides with mitotic preparations were previously incubated with RNase (5 μl RNase 10 mg/ml in 975 μl 2 X SSC). Chromosomal DNA was denatured for 5 min in 70% formamide in 2 X SSC [17.53g of sodium chloride (0,29 M), 8.82 g of sodium citrate and distilled water in a final volume of 1,000 mL, pH 7.0] at 70 °C. The hybridization solution (15 μl formamide to a final concentration of 50%, 6 μl of 50% dextran sulfate, 6 μl of denatured probe, 3 μl of 20 X SSC) was placed onto the slide and incubated in a humid chamber (2 X SSC) at 37 °C overnight. Post hybridization washes were done with 15% formamide at 42° C for 10 min; three washes in 0.1 X SSC at 60 °C for 5 min and 0.5% Tween20 at room temperature for 5 min. For signal detection, slides were immersed in NFDM buffer (20 mL of 20 X SSC, pH7.0; 5 g of powdered skim milk; 80 mL of distilled water) for 15 min; followed by two washes in 5% Tween20 for 5 min at room temperature. The hybridized probes were detected using conjugated FITC-Avidin (Sigma) in C buffer (0.1 M sodium bicarbonate, 0.15 M sodium chloride; pH 7.0) for 60 min. Signal amplification was done with anti-avidin-biotin (1 μl of anti-avidin-biotin and 19 μl of NFDM buffer) incubated in a humid chamber at 37 °C for 15 min, followed by washes with 5% Tween20 and signal detection as described above. The chromosomes were counterstained with DAPI (1,2-diamidin-phenyl-indol) or alternatively with propidium iodide. Slides were mounted with anti-fading reagent Vectashield (Vector).

### Microscopy and Image Processing

Metaphases processed with classical banding methods were photographed in a Zeiss Axioskop microscope using a Canon A640 digital camera, while those subjected to FISH procedures were photographed in an Olympus BX51 coupled with a DP70 CCD camera (Olympus Inc.). The captured images were adjusted for brightness/contrast and the karyotypes were arranged using the software Adobe Photoshop 7.

## Results

**Figure 1:**
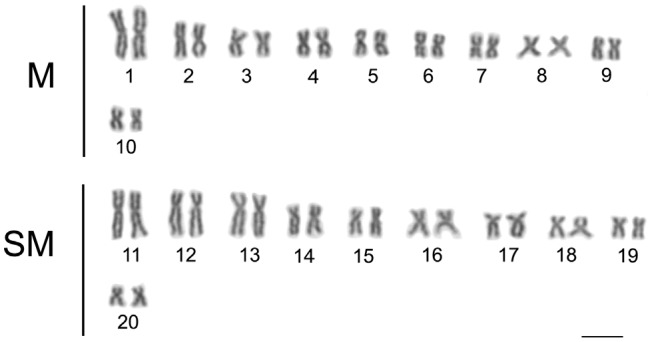
Conventionally stained karyotype of *Hoplias malabaricus*(2n = 40) from the Amazon basin. M = metacentric; SM = submetacentric; Bar = 5 (μm. Specimen ALM-5 (female).

The karyotype of *Hoplias malabaricus* analyzed herein presented a diploid number of 2n = 40 chromosomes, consisting of 20 metacentrics and 20 submetacentrics without evidence of differentiated sexual chromosomes under conventional staining (Figure 1).

**Figure 2:**
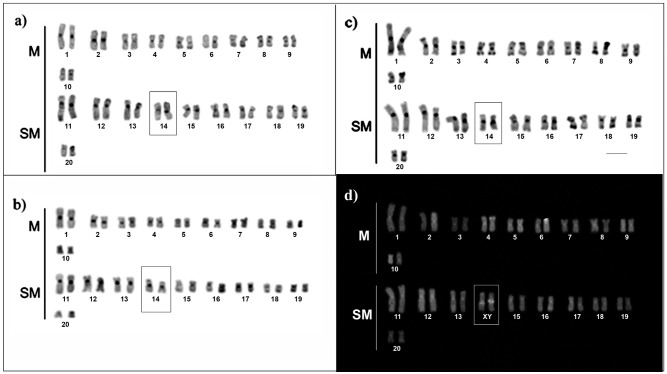
C-banded (a, b, c) and CMA_3_ (d) stained karyotypes of*Hoplias malabaricus* from Amazon basin. The karyotypes are representative of white water (a, d), clear water (b) and black water environments (c). Heteromorphic bands in pair 14 of male karyotypes evidences the nascent XX/XY sexual system. M = metacentric; SM = submetacentric; Bar = 5 (μm. Specimens: a) ALM-5 (female); b) ITA-107 (male); c) 11122 (male), d) AMA-14 (male).

Heterochromatic bands were visualized at the centromere region of all the chromosomes and the telomere region of a few pairs (Figure 2). The heterochromatic material around the telomeric region seemed to be slightly increased in the samples collected from black water (Figure 2c) than in those from white and clear water environments (Figure 2a, b). Minor size variation could be observed in the centromeric heterochromatin of pair 14, which is consistent with male karyotypes (Figure 2 b, c, d). This heteromorphism seems to be associated with unequal accumulation of GC content between homologues of pair 14, as demonstrated by CMA3 staining (Figure 2d).

**Figure 3:**
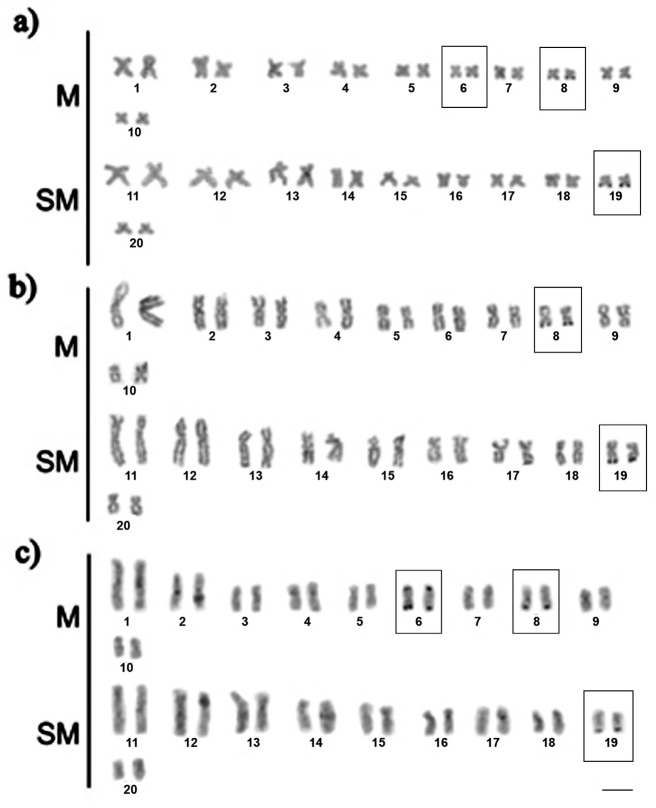
Silver stained karyotypes of *Hoplias malabaricus* from the Amazon basin. The NOR bearing chromosomes are in boxes. The karyotypes are representative of white water (a), clear water (b) and black water environments (c). M = metacentric; SM = submetacentric; Bar = 5 (μm. Specimen: a) ALM-5 (female); b) JUA-13 (female); c) 11133 (male).

Multiple NORs were visualized on distal portions of chromosomes 6, 8 and 19 (Figure 3), however, slight variation could be evidenced when samples from the distinct environments were compared. In this way, karyotypes representative of white water and black water either showed NORs on the chromosomes 6, 8 and 19, but a bitelomeric NOR on chromosome 6, which was an exclusive trait of samples from black water (Figure 3c). In contrast to the observed in specimens from white and black water environments, samples from clear water river did not show NORs on chromosome 6 (Figure 3b).

**Figure 4:**
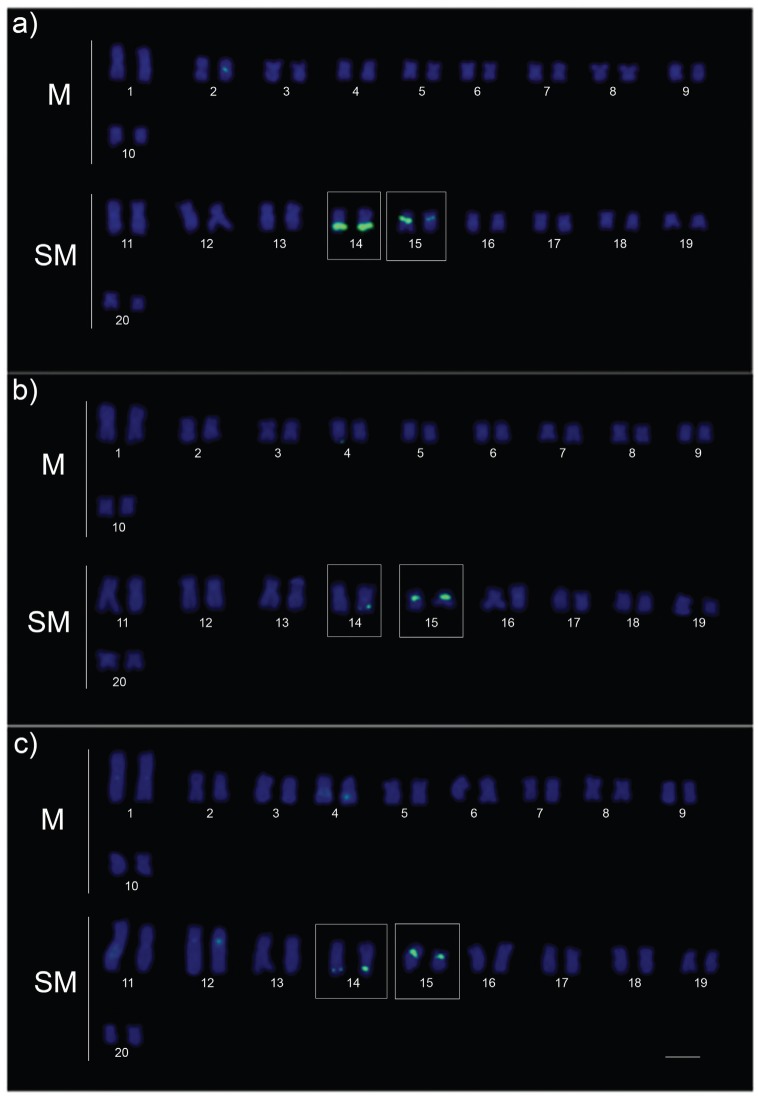
Localization of rDNA 5S repeats (green) in the karyotypes of*Hoplias malabaricus* from the Amazon basin, cytotype C. The labeled chromosomes are shown in boxes. The karyotypes are representative of white water (a), clear water (b) and black water environments (c). M = metacentric; SM = submetacentric; Bar = 5 (μm. Specimens: a) AMA-14 (male); b) JUA-14 (male); c) 10411 (male).

**Figure 5:**
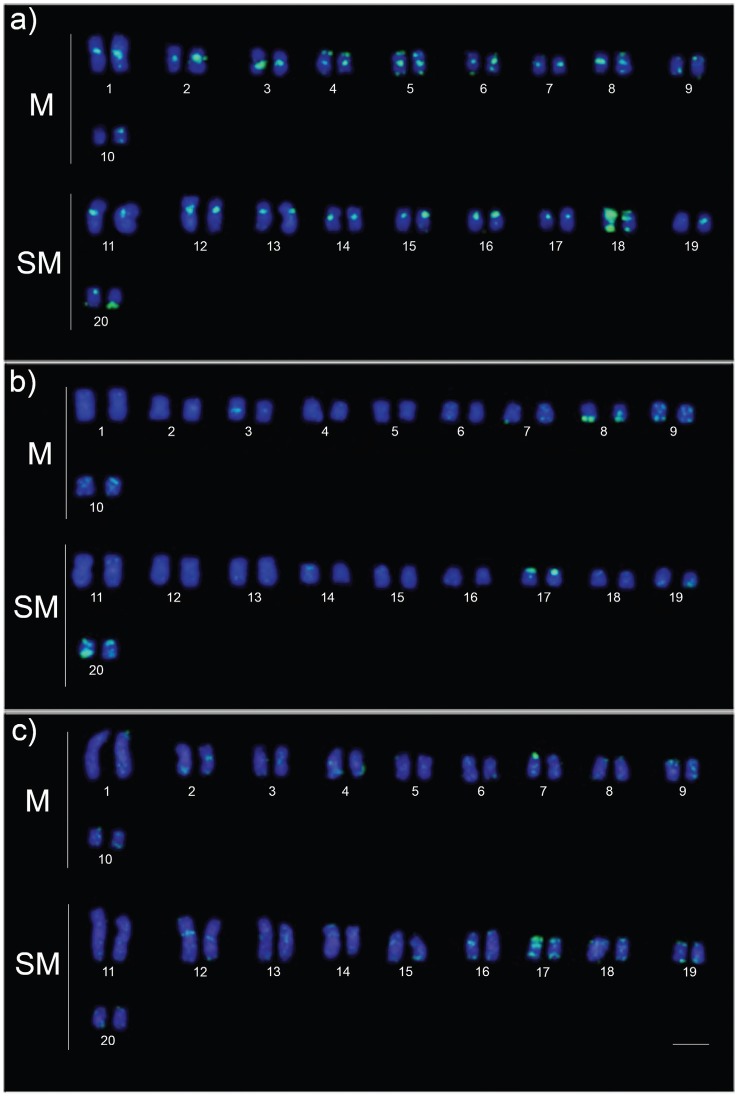
Hybridization of *Rex3* probes on karyotype of*Hoplias malabaricus* (cytotype C) from the Amazon basin. The karyotypes are representative of white water (a), clear water (b) and black water environments (c). M = metacentric; SM = submetacentric; Bar = 5 (μm. Specimens: a) AMA-14 (male); b) JUA-14 (male); c) 10411 (male).

*rDNA-5S* probes yielded conspicuous fluorescent signals on the chromosomes 14 and 15 (Figure 4). Variation in spot size of the hybridization signal was clearly observed between the homologs of the pair 15 in samples from white water and the pair 14 in those from clear water (Figure 4a, b). Probes of the retroelement*Rex3* showed marked differences between karyotypes of fishes from white water compared to ones from clear and black water. The first exhibited stronger fluorescent signals associated with regions of centromeric heterochromatin, while the latter showed faint signals in a few chromosomes (Figure 5).

## Discussion

It has been well demonstrated that geology, water physical-chemical properties and biological features are responsible for the large heterogeneity of aquatic environments from the Amazon basin (Val and Almeida-Val, 1995; Goulding *et al.*, 2003). This environmental variability plays a role in the local adaptive processes of aquatic life, including an array of morphological, physiological and probably genetic adaptations (Junk *et al.*, 1989).

Cytogenetic markers proved to be important tools to demonstrate variability among*Hoplias malabaricus* populations (Bertollo *et al.*, 2000; Santos *et al.*, 2009; Jacobina *et al.*, 2011). Based on the similarities observed in diploid number and the gross chromosomal morphology we recognized the karyotype of *H. malabaricus* (2n = 40) from the Amazon basin in the present study as a representative of cytotype C, as defined in Bertollo *et al.* (2000). However, when compared with cytotype C population, from the Bento Gomes river, Mato Grosso (Cioffi *et al.*, 2009a), we detected variation in the karyotypic formulae and NOR labeling sites. Such geographic variation in the macrostructure of cytotype C could be resultant of chromosome rearrangements like pericentric inversions leading to transformation of metacentric into submetacentric form or vice-versa.

Despite of diverging from the Bento Gomes river population in term of gross chromosomal morphology (karyotypic formulae), the *H. malabaricus*populations from the Amazon basin (present study) preserve signatures of the initial steps toward a nascent sexual chromosome system XX/XY, as suggested in Cioffi and Bertollo (2010). Based on karyotypic comparisons and analyses of C-bands and CMA3 stained metaphases we assume a homology between the sexual chromosome pair XX/XY (pair 11) previously detected (Cioffi and Bertollo, 2010) and pair 14 (Figure 2), recognized herein, as sexual chromosomes in the cytotype C from the Amazon basin.

Multiple NORs are a common feature of the *Hoplias malabaricus* genome (Born and Bertollo, 2006; Vicari *et al.*, 2006; Santos *et al.*, 2009; Cioffi *et al.*, 2009a, b). In the specimens analyzed herein were observed a reduction in the number of NOR bearing chromosomes compared to samples from the Bento Gomes river (Cioffi *et al.*, 2009a). Since the populations of cytotype C are largely distributed across South America (Bertollo *et al.*, 2000) and their sedentary behavior may favor to reduce gene flow and promote divergence (Blanco *et al.*, 2010), minor variations within the cytotype C representatives could be expected. Karyotypic differences, including NOR polymorphism, have been recorded in populations of cytotype A, another broadly dispersed cytotype of *H. malabaricus* (Born and Bertollo, 2001; Vicari*et al.*, 2005; Cioffi*et al.*, 2009a; Blanco*et al.*, 2010).

The location of 5S rDNA repeats seems to be conserved among the samples from distinct habitats (white, clear and black waters), but the spot size variation observed in chromosomes 14 and 15 revealed a heteromorphism of this genome region. This phenomenon can be explained by unequal exchange events driving to increase/decrease copy number between homologs (Martins, 2007). Moreover, the accumulation of distinct classes of repetitive DNA may play a role in the differentiation of sexual chromosomes (Cioffi and Bertollo, 2010).

Abundance of *Rex3* retroelement repeats was larger in the samples originating from white water habitat, resembling a compartment fashion of cytogenomic organization. On the other hand, the pattern visualized in karyotypes from clear and black water habitats was interpreted as a dispersed mode. This retroelement has been commonly detected on fish genomes and usually hybridizes in a dispersed mode through all the chromosomes (Ozouf-Costaz *et al.*, 2004; Ferreira *et al.*, 2010), but sometimes it accumulates on specific chromosomes or chromosomal region, as was observed in Y chromosome of *Chionodraco hamatus* (Ozouf-Costaz *et al.*, 2004) and centromeres of*Astronotus ocellatus* (Mazzuchelli and Martins, 2009).

Transposable elements can respond to changes in cellular and external environment (Capy *et al.*, 2000; Casacuberta and Gonzalez 2013). Vieira and Biémont (1996) evidenced a copy number increase of *Drosophila* 412 element in response to temperature decrease. Herein we detected clear evidence of copy number variation in distinct types of repetitive DNA in the cytogenome of *Hoplias malabaricus* from white, black and clear water rivers of the Amazonian basin. This result leads to infer a possible mechanism of adaptation by enrichment of *Rex3* in *Hoplias malabaricus* in these different habitats due to stress caused by changes in water properties in the past (Casacuberta and Gonzalez, 2013) that maintained this accumulation of retroelement based on the sedentary habits of these fish.

Therefore, we conclude that slight differences observed in *Hoplias malabaricus* may reflect a fine mechanism of cytogenomic shaping, and possibly represents a signature of ongoing local adaptation processes, since the populations studied herein inhabit markedly distinct aquatic environments. Further studies are needed to explore this hypothesis and shed light on how the architecture of fish genomes responds to environmental changes.
